# A polymer dataset for accelerated property prediction and design

**DOI:** 10.1038/sdata.2016.12

**Published:** 2016-03-01

**Authors:** Tran Doan Huan, Arun Mannodi-Kanakkithodi, Chiho Kim, Vinit Sharma, Ghanshyam Pilania, Rampi Ramprasad

**Affiliations:** 1 Institute of Materials Science, University of Connecticut, 97 North Eagleville Rd., Unit 3136, Storrs, Connecticut 06269, USA; 2 Materials Science and Technology Division, Los Alamos National Laboratory, Los Alamos, 87545, New Mexico USA

**Keywords:** Electronic properties and materials, Computational chemistry, Density functional theory, Atomistic models

## Abstract

Emerging computation- and data-driven approaches are particularly useful for rationally designing materials with targeted properties. Generally, these approaches rely on identifying structure-property relationships by learning from a dataset of sufficiently large number of relevant materials. The learned information can then be used to predict the properties of materials not already in the dataset, thus accelerating the materials design. Herein, we develop a dataset of 1,073 polymers and related materials and make it available at http://khazana.uconn.edu/. This dataset is uniformly prepared using first-principles calculations with structures obtained either from other sources or by using structure search methods. Because the immediate target of this work is to assist the design of high dielectric constant polymers, it is initially designed to include the optimized structures, atomization energies, band gaps, and dielectric constants. It will be progressively expanded by accumulating new materials and including additional properties calculated for the optimized structures provided.

## Background & Summary

A central tenet of data-driven materials discovery is that if the volume of accumulated or available data is sufficiently large, and if it can be mined properly with suitable data-driven techniques, the process of designing a new material could be more efficient and rational^1–11^. This notion has lead to the development of many useful materials databases^12–18^. The present contribution deals with polymeric materials. Given the complexity of the chemical and configurational/morphological space of polymeric materials, the creation of a database focusing on this materials class is challenging. Nevertheless, if systematic steps can be taken in this direction, consistent with the charter of the Materials Genome Initiative, we will progressively get closer to the rational design and discovery of application-specific polymers.

Within this context, it is worth noting that the recent rational development of nearly a hundred novel polymeric dielectrics for capacitive or electrostatic energy storage^19–26^ has benefitted from the synergy between experimental and computational efforts, of which computations at various levels, including force fields^27–30^ and density functional theory (DFT)^31,32^, have provided critical guidance. Given a polymer chemical composition, the computational step mainly involves predicting the lowest-energy structures and computing the associated dielectric constant ε and band gap *E*_g_. Those with high ε and high *E*_g_ were then identified, leading to the experimental realizations of polymers with desired performances such as high energy density, low loss, etc., refs 19–26.

This contribution describes a dataset of 1,073 polymers and related materials as the first step aiming at the rational design of polymers by data-driven approaches. The dataset reported herein, referred to as ‘‘polymer dataset’’ for convenience, was prepared at a uniform and consistent level of first-principles DFT computations. Since our initial goal is to assist the design of high dielectric constant polymers for energy storage, the polymer dataset supplies the equilibrium (relaxed) structures of the materials associated with relevant calculated properties, including the atomization energy ε_at_, the dielectric constant ε and the energy band gap *E*_g_. The initial structures used for the preparation were collected either from other available sources or, quite often, from computational structure searches. This dataset, which is available at http://khazana.uconn.edu/, can readily be expanded in multiple ways, i.e., new properties can be calculated from the provided equilibrium structures, and new materials with relevant calculated properties can also be progressively added. Furthermore, it may also serve as a playground for data-mining.

## Methods

### Workflow

**Figure 1:**
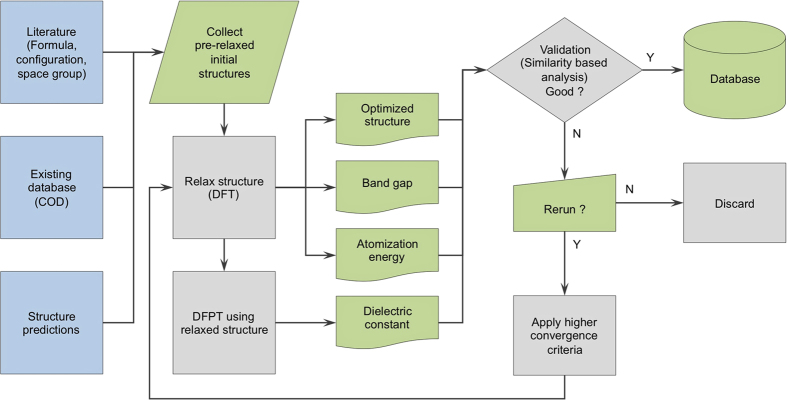
Scheme for preparing the dataset of polymers and related materials. USPEX and minima-hopping are two structure prediction methods that were used for generating a majority of the dataset.

The workflow in Fig. 1 summarizes the preparation of the polymer dataset. In the first step, crystal structures of polymers and related compounds were collected from various available sources, including the reported literature, the *Crystallography Open Database* (COD)^15^, and our structure prediction works^20–26^. Those obtained from structure prediction runs were subjected to a preliminary filter (described below), removing any obvious redundancy of identical structures. Then, the selected structures were optimized by DFT calculations, yielding the equilibrium structures and their atomization energies ε_at_. The energy band gap *E*_g_ was then calculated on a dense grid of **k** points while their dielectric constant ε, which is composed of an electronic part ε_elec_ and an ionic part ε_ion_, was computed within the framework of density functional perturbation theory (DFPT)^33^. In the next step, the computational scheme and the calculated results were validated with available measured data, including the measured band gap *E*_g_, the dielectric constant ε and/or the infrared spectroscopy (IR) measurements. Those which do not agree with the available experimental data are subjected to further calculations at tighter convergence criteria of residual atomic force (see Technical Validation for more details), and if better agreement is not reached, these points are removed from the dataset. A post-filtering step was finally performed on the whole dataset, keeping only distinct data points. Relaxed structures of all the materials are finally converted into the crystallographic information format (cif) using the pymatgen library^34^. A note was also provided together with the dataset, indicating the convergence criteria of the datapoints reported herein.

### Structure accumulation

Our dataset includes three primary subsets, each of them originating from a distinct source. Subset 1 consists of *common polymers* which have already been synthesized, resolved, and reported elsewhere. This set contains 34 polymers, listed in Table 1. Collecting polymer structures of this class is challenging because the reported data is widely scattered, and in case the information obtained is sufficient to reconstruct structures, this work has to be done manually and hence, substantially laborious. We further note that only for a few of them, measurement for band gap, dielectric constant, and/or infrared (IR) spectrum have been performed. This data was used for the validation step.

**Figure 2:**
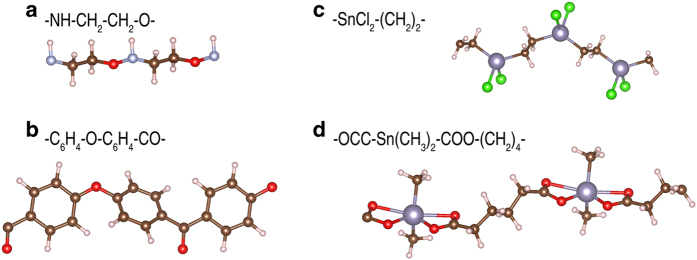
Organic polymer chains with repeat units of –NH–CH_2_–CH_2_–O– (**a**) and –C_6_H_4_–O–C_6_H_4_–CO– (**b**) and organometallic polymer chains with repeat units of –SnCl_2_–(CH_2_)_2_– (**c**) and –OOC–Sn–(CH_3_)_2_–COO–(CH_2_)_4_– (**d**). Carbon, hydrogen, oxygen, nitrogen, chlorine, and tin atoms are shown in dark brown, light pink, red, light cyan, green, and dark cyan, respectively.

Subset 2 includes 314 new organic polymers (284 of them have been used in ref. 11) and 472 new organometallic polymers. Their structures were generated from a computation-driven strategy^19,20^ which has been used to rationally design various classes of polymeric dielectrics^11,20–26^. The starting point of this strategy is a pool of common polymer building blocks, which are either organic, e.g., –CH_2_–, –NH–, –CO–, –O–, –CS–, –C_6_H_4_–, and –C_4_H_2_S–, or inorganic (metal-containing) like –COO–Sn(CH_3_)_2_–OCC–, –SnF_2_–, and –SnCl_2_–. The repeat unit of an organic polymer is then created by concatenating a given number of organic building blocks while that of an organometallic polymer contains at least one inorganic block linked with a chain of several CH_2_ groups. Next, chains of the repeat units (illustrated in Fig. 2) are packed in low-energy crystal structures which are determined by Universal Structure Predictor: Evolutionary Xtallography (USPEX)^23,35^ or minima-hopping (MH)^36,37^, two of the currently most powerful structure prediction methods. In brief, these methods allow for predicting the low-energy structures of a material as the local minima of the potential energy surface, constructed from DFT energy. The efficiency of these methods have been successfully demonstrated for many different materials classes^38–41^, including a large number of organic^20,23^ and organometallic polymers^24–26^.

For each structure prediction run, the lowest-energy structure and those within 200 meV per atom above it were collected. The number of structures within this energy window is material-dependent, ranging from several to several dozens. Because many of them are just slightly different by small perturbations in the atomic arrangement, a preliminary filtering step was used to remove this redundancy. In particular, we used a clustering algorithm (hierarchical) to group those which are different by less than 5 meV per atom in ε_at_ and less than 0.1 eV in *E*_g_, keeping the representative structures. Only those with polymeric motifs, when visually confirmed, are selected for the next steps. In the predicted polymer structures, especially for those of organometallic polymers, these polymeric chains are not necessarily isolated, i.e., inter-chain bonds may occur in various fashions^24–26^.

The material structures used to prepare subset 3 were collected from COD. Generally, materials provided by COD are not polymers, but a number of them are collected in this dataset as they are closely related to the examined polymers. Although collecting materials structures from this database is straightforward, we limited ourselves to only those whose cell volumes are not too large, i.e., roughly 1,500 Å^3^ and below. This subset contains 253 molecular organic and organometallic crystals, 178 of them have recently been used in ref. 10 by some of us.

Table 2 summarizes the contents of the polymer dataset, which contains both polymers (subset 1 and 2) and non-polymers (subset 3). In terms of chemistry, the included materials can be classified as either organic or organometallic, incorporating different metals in their backbone. The complete list of chemical elements that appear in this dataset is given in Table 3.

### Numerical calculations

The computed data reported in our dataset was prepared with density functional theory (DFT)^31,32^ calculations, using the projector augmented-wave (PAW) formalism^42^ as implemented in Vienna *Ab initio* Simulation Package (vasp)^43–46^. The default accuracy level of our calculations is ``Accurate'', specified by setting PREC=Accurate in all the runs with vasp. The basis set includes all the plane waves with kinetic energies up to 400 eV, as recommended by vasp manual for this level of accuracy. PAW datasets of version 5.2, which were used to describe the ion-electron interactions, are also summarized in Table 3. The van der Waals dispersion interactions, known^47^ to be important in stabilizing soft materials dominated by non-bonding interactions like polymers^48^, were estimated with the non-local density functional vdW-DF2 (ref. 49). The generalized gradient approximation (GGA) functional associated with vdW-DF2, i.e., refitted Perdew-Wang 86 (rPW86)^50^, was used for the exchange-correlation (XC) energies.

Because the examined material structures are significantly different in terms of the cell shape, the sampling procedure of their Brillouin zones must be handled appropriately. For each structure, a Monkhorst-Pack **k**-point mesh^51^ of a given spacing parameter *h*_k_ in the reciprocal space was used. For the geometry optimization and dielectric constant calculations, *h*_k_=0.25 Å^−1^ while the band gap calculations have been performed on a finer Γ-centered mesh with *h*_k_=0.20 Å^−1^. We further set the lower limit for the Monkhorst-Pack mesh dimensionality, that is, the number of grid points along any reciprocal axis is no less than 3, regardless of how short the reciprocal lattice dimension along this axis is.

During the relaxation step, we optimized both the cell and the atomic degrees of freedom of the materials structures until atomic forces are smaller than 0.01 eV Å^−1^. Calculations for band gap *E*_g_ was then carried out on top of the equilibrium structures. Because *E*_g_ is typically underestimated with a GGA XC functional like rPW86 (ref. 52), this important physical property has also been calculated with the hybrid Heyd-Scuseria-Ernzerhof (HSE06) XC functional^53,54^ with an expectation that the calculated result would become much closer to the true material band gap. Both *E*_g_^GGA^ and *E*_g_^HSE06^, the band gap calculated at the GGA-rPW86 and HSE06 levels of theory, are provided in all the entries of the dataset (see File format for more details). Finally, the dielectric constant ε of these structures was calculated within the DFPT formalism as implemented in vasp package. Calculations of this type involve the determination of the lattice vibrational spectra at Γ, the center of the Brillouin zone. This information is also used to compute the IR spectra of some structures for the purpose of validation.

### Post-filtering

Given that the sources of the polymer dataset reported herein are diversified, any clear duplicate and/or redundancy should be identified and removed. Because the preliminary filtering step was performed only on subset 2 based on their DFT energy and band gap estimated during the structure prediction runs with a limited accuracy, an additional filtering step was performed on the whole dataset. Within this step, all cases with the same chemical composition but different by less than 0.1 eV in *E*_g_, less than 5 meV per atom in ε_at_, and less than 0.1 in both ε_elec_ and ε_ion_, are clustered. At this point, the number of clustered points is not large, and all of them were inspected visually, keeping only distinct materials.

## Data Records

The complete dataset of 1,073 polymers and related materials can be downloaded as a tarball from Dryad Repository (Data Citation 1) or can be accessed via http://khazana.uconn.edu/ (all the records with ID from 0001 to 1073). All 4,292 DFT runs of the entire dataset (for each structure, there are 4 runs, including relax, dielectric, GGA band gap, and HSE06 band gap) are hosted by NoMaD Repository (Data Citation 2).

### File format

All the information reported in the dataset for a given material is stored in a file, named as 0001.cif, where a cardinal number (0001 in this example) is used for the identification of the entry in the dataset. The first part of a file of this type is devoted to the optimized structure in the standard cif format which is compatible with majority of visualization software. Other information, including the calculated properties, is provided as the comments lines in the second part of the file as follow

# Source: VSharma_etal:NatCommun.5.4845(2014)

# Class: organic_polymer_crystal

# Label: Polyimide

# Structure prediction method used: USPEX

# Number of atoms: 32

# Number of atom types: 4

# Atom types: C H O N

# Dielectric constant, electronic: 3.71475E+00

# Dielectric constant, ionic: 1.54812E+00

# Dielectric constant, total: 5.26287E+00

# Band gap at the GGA level (eV): 2.05350E+00

# Band gap at the HSE06 level (eV): 3.30140E+00

# Atomization energy (eV/atom): -6.46371E+00

# Volume of the unit cell (A^3): 2.79303E+02

While most of the keywords are clear, we used Source to provide the origin of the material structure and Class to refer to the class of materials which can either be ‘‘organic polymer crystal’’, ‘‘organometallic polymer crystal’’, ‘‘organic molecular crystal’’, or ‘‘organometallic molecular crystal’’. Keyword Label was used to provide more detailed information on the material, which can be the common name of the material if it is available, the ID of the record obtained from COD, or the repeat unit of the polymer structure predicted.

### Graphical summary of the dataset

**Figure 3:**
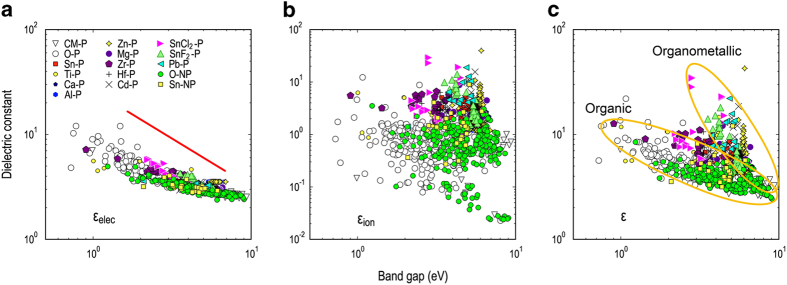
A summary of the polymer dataset based on the calculated band gap $E_{g}^{\mathrm{HSE06}}$ and the dielectric constants $\varepsilon_{\mathrm{elec}}$ (**a**), $\varepsilon_{\mathrm{ion}}$ (**b**), and $\varepsilon =\varepsilon_{\mathrm{elec}}+\varepsilon_{\mathrm{ion}}$ (**c**). In the figure keys, ‘‘CM’’, ‘‘P’’, ‘‘NP’’, and ‘‘O’’ refer to ‘‘Common’’, ‘‘Polymer’’, ‘‘Non-Polymer’’, and ‘‘Organic’’, respectively. For organometallic polymers, the identity of the metal element included is used. The polymers developed by the structure prediction based pathway in refs 19–26 are labeled as ‘‘Dev-P’’.

To graphically summarize the polymer dataset, we visualize it in the property space. Because the band gap and the dielectric constant are the primary properties reported by this dataset, three plots, namely $E_{g}^{\mathrm{HSE06}}-\varepsilon_{\mathrm{elec}}$, $E_{g}^{\mathrm{HSE06}}-\varepsilon_{\mathrm{ion}}$, and $E_{g}^{\mathrm{HSE06}}-\varepsilon$, were compiled and shown in Fig. 3. Materials from different classes are shown in different colors to clarify the role of the polymer chemical composition in controlling *E*_g_ and ε. Within the recent effort of developing polymers for high-energy-density applications^19–26^, such plots are useful for identifying promising candidates, i.e., those which have high dielectric constant while maintaining sufficient band gap (*E*_g_≥3 eV).

Figure 3a clearly indicates a limit of the form $\varepsilon_{\mathrm{elec}}∼1/E_{g}$ between $\varepsilon_{\mathrm{elec}}$ and *E*_g_, which is applicable for both organic and organometallic classes of materials. We note that this behavior has also been reported elsewhere^10,19^. Figure 3c, on the other hand, demonstrates that the classes of organic and organometalic polymers and molecular crystals occupy different regions in the property space. At a given value of band gap, the organometallic polymers are generally much higher than the organic polymers in terms of the dielectric constant. While a fairly large number of organometallic polymers were already developed^24–26^, this observation suggests that there remains significant room for manipulating the dielectric constant of the organometallic polymers.

## Technical Validation

**Figure 4:**
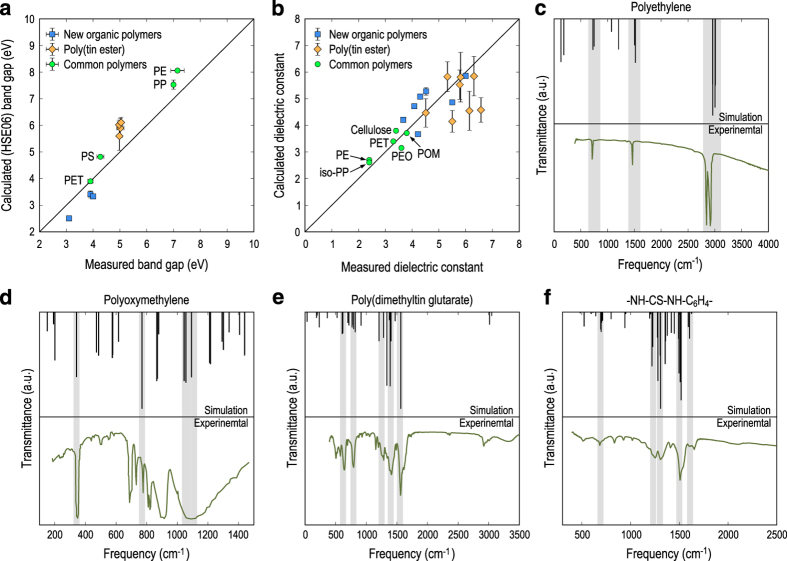
Calculated and measured dielectric constants of (**a**) several inorganic compounds, and (**b**) the polymers reported in refs 20–22 (new organic polymers) and refs 24–26 (poly(tin ester)). The error bars originated from different (energetically competing) structures predicted for a given polymer. For organometallic polymers, the error bars are significant due to the diversity of structural motifs involving the aforementioned inter-chain bonds, which are not present in organic polymers. In (**c**), (**d**), (**e**), and (**f**), the simulated and measured infrared spectra of orthorhombic polyethylene, orthorhombic polyoxymethylene, poly(dimethyltin glutarare), and polythiourea are shown. The experimental data of these three polymers was taken from refs 60,55, 24, 20, respectively. Shadow areas are given to indicate the agreement between simulated and measured transmitance peaks.

Among the materials properties reported in the present dataset, the atomization energy $E_{\mathrm{at}}$ is physically relevant and has always been used as a standard method for examining the thermodynamic stability of various classes of materials, including inorganic crystals^38–41^ and polymers^19–26^. While the band gap $E_{g}^{\mathrm{GGA}}$ calculated at the GGA level of DFT is not ready to be compared with the measured data due to the aforementioned well-known underestimation^52^, $E_{g}^{\mathrm{HSE06}}$ (the band gap calculated with the HSE06 XC functional) is expected to be rather close to the true band. We show in Fig. 4a $E_{g}^{\mathrm{HSE06}}$ of 11 polymers for which the band gap has been measured experimentally. The calculated band gap seems to agree pretty well with the measured data with a numerical discrepancy of about 20% and below.

We now consider the calculations of the dielectric constants, namely $\varepsilon_{\mathrm{elec}}$ and $\varepsilon_{\mathrm{ion}}$. Overall, the theoretical foundations and the implementations for calculating $\varepsilon_{\mathrm{elec}}$ and $\varepsilon_{\mathrm{ion}}$ are well developed and tested, leading to rather accurate results. Within the DFT-based perturbative approach, $\varepsilon_{\mathrm{elec}}$ is computed via the response to the external field perturbations while $\varepsilon_{\mathrm{ion}}$ is evaluated through the phonon frequencies at the Γ point of the Brillouin zone. To be precise, the dielectric response of a crystalline insulator to an external electric field **E** is given in terms of a frequency-dependent tensor $\varepsilon^{\alpha \beta }(\omega )$. To linear order, the electronic contribution of the dielectric tensor is given by

(1)εelecαβ(ω)=1+4π∂Pα∂Eβ,

where *P*_*α*_ is the component along the *α* direction of the induced polarization **P**. On the other hand, the ionic part of the dielectric tensor is determined as

(2)εionαβ(ω)=4πΩ∑mSmαβωm,q=02−ω2.

In this expression, Ω is the volume of the simulation cell, appearing as a normalization factor. The sum is taken over the index *m* of the phonon normal modes, which assumes the frequency *ω*_*m*,**q**=0_ at the Brillouin zone center (**q**=0) while the mode oscillator strength *S*_*mαβ*_ is determined through the Born effective charge *Z*_*s*,αβ_* of the atom *s*. For an isotropic material, the dielectric constant of the practical interest is taken to be the average value of its diagonal elements at the static limit, i.e., $\varepsilon =\frac{1}{3}\sum_{\alpha }[\varepsilon^{\alpha \alpha }(\omega \to 0)]$.

Equation 2 implies that at the limit of *ω*→0, $\varepsilon_{\mathrm{ion}}^{\alpha \beta }(\omega )$ is rather sensitive to the numerical accuracy of *ω*_*m*,**q**=0_, which, in turn, suggests highly equilibrated materials structures for the DFPT calculations. As mentioned in the Workflow Section, if the calculated dielectric constant ε of a polymer is different from its measured data (this information is available for just a limited number polymers in subset 1 and 2) by more than 20%, the structures are further optimized until the residual atomic forces are smaller than 0.001 eV Å^−1^. Only those with calculated dielectric constant within 20% of the experimental data [shown in Fig. 4b] are kept.

Within our dataset, the IR spectrum was measured for some materials. From the computational side, this material characteristic can also be calculated rather accurately from the byproducts of the dielectric constant calculations with DFPT. In particular, the intensities of the infrared-active modes are given by^56^

(3)Im∝∑α|∑sβZ*s,αβ*em,sβ|2,

where *e*_*m*,*sβ*_ is the *β* component of the normalized vibrational eigenvector of the mode *m* at the atom *s*. Obviously, all of the necessary quantities needed to calculate *I*_*m*_ according to Equation 3 can be obtained within the DFPT-based computational scheme of ε, thus requiring essentially no computational overhead. This approach has widely been used in characterizing various classes of materials^57,58^. We show in Figure 4c–f the IR spectra calculated for four polymers, including orthohombic polyethylene, orthohombic polyoxymethylene, poly(dimethyltin glutarate)^24^, and polythiourea^20^, each of them is compared with the corresponding measured IR spectrum. The excellent agreement between the calculated and the measured IR spectra can be regarded as a supportive validation of the computational scheme based on DFT calculations used for this polymer dataset.

## Usage Notes

This dataset, which includes a variety of known and new organic and organometallic polymers and related materials, has been consistently prepared using first-principles calculations. While the HSE06 band gap $E_{g}^{\mathrm{HSE06}}$ is believed to be fairly close to the true band gap of the materials, the GGA-rPW86 band gap is also reported for completeness and for further possible analysis. The reported atomization energy and the dielectric constants are also expected to be accurate.

The polymer dataset is one among many recently developed datasets which can be used for designing materials by various data-driven approaches. To be specific, this dataset is expected to be useful in the development of polymers for energy storage and electronics applications. Moving forward, the development of this dataset will be continuously validated and updated, and the most recent version can be accessed at repository http://khazana.uconn.edu/.

## Supplementary Material



## Figures and Tables

**Table 1:** List of the common polymers summarized in this dataset and the corresponding references.

**Polymer**	**Ref.**	**Polymer**	**Ref.**
Polyethylene	^61^	Isotactic polypropylene	^62^
Polyethylene oxide	^23^	Polyglutamic acid	^23^
Cellulose	^23^	Poly(1,1,2-trifluoroethene)	^63^
Clathrate syndiotactic polystyrene	^64^	Poly(2,5-dihydrothiophene-2,5-diyl)	^65^
Poly ε-caprolactone	^66^	Poly(2,6-benzothiazole)	^67^
Poly(3,3,3-trifluoro-2-methyloxirane)	^68^	Poly(ethene-alt-hexafluoroacetone)	^69^
Polyethylene adipate	^70^	Polyethylene suberate	^70^
Polyoxymethylene	^23^	Poly(p-phenylene oxide)	^23^
Poly(p-phenylene sulfide)	^71^	Poly(propylene sulfide)	^72^
Poly(p-xylylene)	^23^	Poly-tetrafluoroethylene-alt-ethylene	^73^
Poly(tetramethylene terephthalate)	^74^	Poly(trimethylene sebacate)	^75^
Poly(vinyl fluoride)	^76^	Polyethylene terephthalate	^77^
Polyvinylidene fluoride (delta)	^78^	Polyvinylidene fluoride (beta)	^78^
Syndiotactic polypropylene	^79^	Poly(2,5-benzoxazole)	^67^
Poly(2-vinylpyridine)	^80^	Polyacrylonitrile	^81^
Polyglycine	^82^	Poly (m-phenylene isophthalamide)	^83^
Poly(m-pyridine)	^84^	Poly(p-phenylene benzobisoxazole)	^85^

**Table 2:** Summary of the data subclasses in the polymer dataset.

**ID**	**No. of points**	**Descriptions**	**Reference**
0001–0034	34	Common polymers	^23,61–85^
0035–0348	314	New organic polymers	^11,20,21,23^
0349–0410	62	Poly(tin ester)	^24–26^
0411–0447	37	Titanium containing polymers	
0448–0460	13	Calcium containing polymers	
0461–0470	10	Aluminum containing polymers	
0471–0572	102	Zinc containing polymers	
0573–0588	16	Magnesium containing polymers	
0589–0610	22	Zirconium containing polymers	
0611–0630	20	Hafnium containing polymers	
0631–0741	111	Cadmium containing polymers	
0742–0763	22	SnCl_2_ containing polymers	
0764–0796	33	SnF_2_ containing polymers	
0797–0820	24	Lead containing polymers	
0821–0854	34	Molecular crystals of C and H	^10,15^
0855–0998	144	Molecular crystals of C, H, & O	^10,15^
0999–1050	52	Molecular crystals of C, H, N, & O	^15^
1051–1073	23	Molecular crystals of C, H, O, & Sn	^15^

**Table 3:** VASP PAW potentials of the elements used for calculations in this work.

**Element**	**POTCAR**	**Element**	**POTCAR**	**Element**	**POTCAR**
Aluminum	Al	Bromine	Br	Carbon	C
Calcium	Ca_sv	Cadmium	Cd	Chlorine	Cl
Fluorine	F	Hydrogen	H	Hafnium	Hf_sv
Magnesium	Mg_sv	Nitrogen	N	Oxygen	O
Phosphorus	P	Lead	Pb_d	Sulfur	S
Tin	Sn_d	Titanium	Ti_sv	Zinc	Zn
Zirconium	Zr_sv				
